# Longer Telomere Length in Peripheral White Blood Cells Is Associated with Risk of Lung Cancer and the rs2736100 (*CLPTM1L-TERT*) Polymorphism in a Prospective Cohort Study among Women in China

**DOI:** 10.1371/journal.pone.0059230

**Published:** 2013-03-26

**Authors:** Qing Lan, Richard Cawthon, Yutang Gao, Wei Hu, H. Dean Hosgood, Francesco Barone-Adesi, Bu-Tian Ji, Bryan Bassig, Wong-Ho Chow, Xiaoou Shu, Qiuyin Cai, Yongbin Xiang, Sonja Berndt, Christopher Kim, Stephen Chanock, Wei Zheng, Nathaniel Rothman

**Affiliations:** 1 Division of Cancer Epidemiology and Genetics, National Cancer Institute, NIH, DHHS, Bethesda, Maryland, United States of America; 2 Department of Human Genetics, University of Utah, Salt Lake City, Utah, United States of America; 3 Department of Epidemiology, Shanghai Cancer Institute, Shanghai, Peoples Republic of China; 4 Division of Epidemiology, Department of Medicine, Vanderbilt University Medical Center and Vanderbilt-Ingram Cancer Center, Nashville, Tennessee, United States of America; The University of Texas M. D. Anderson Cancer Center, United States of America

## Abstract

A recent genome-wide association study of lung cancer among never-smoking females in Asia demonstrated that the rs2736100 polymorphism in the *TERT-CLPTM1L* locus on chromosome 5p15.33 was strongly and significantly associated with risk of adenocarcinoma of the lung. The telomerase gene *TERT* is a reverse transcriptase that is critical for telomere replication and stabilization by controlling telomere length. We previously found that longer telomere length measured in peripheral white blood cell DNA was associated with increased risk of lung cancer in a prospective cohort study of smoking males in Finland. To follow up on this finding, we carried out a nested case-control study of 215 female lung cancer cases and 215 female controls, 94% of whom were never-smokers, in the prospective Shanghai Women’s Health Study cohort. There was a dose-response relationship between tertiles of telomere length and risk of lung cancer (odds ratio (OR), 95% confidence interval [CI]: 1.0, 1.4 [0.8–2.5], and 2.2 [1.2–4.0], respectively; P trend = 0.003). Further, the association was unchanged by the length of time from blood collection to case diagnosis. In addition, the rs2736100 G allele, which we previously have shown to be associated with risk of lung cancer in this cohort, was significantly associated with longer telomere length in these same study subjects (P trend = 0.030). Our findings suggest that individuals with longer telomere length in peripheral white blood cells may have an increased risk of lung cancer, but require replication in additional prospective cohorts and populations.

## Introduction

Telomerase gene *TERT* is a reverse transcriptase that is critical for telomere replication and stabilization by controlling telomere length. Telomeres are DNA–protein complexes that cap the ends of chromosomes and promote chromosomal stability. To date, the associations between telomere length and cancer risk are inconclusive. Most initial studies used a case-control design and reported that shorter telomere length measured in peripheral white blood cells was associated with increased risk of cancer [1]. In contrast, some recent publications using a prospective cohort design have suggested that longer telomere length may be associated with increased risk of certain tumors, including lung, lymphoma, hepatocellular carcinoma, and melanoma [2]–[5]. Recently, two case-control studies reported that longer telomere length was associated with colorectal, breast cancer, and breast cancer survival [6]–[9]. Shorter telomere length has been associated with aging and both shorter and longer telomere length have been associated with risk of a number of chronic diseases [10], [11], although this heterogeneity may be explained in part by case-control vs. prospective cohort study designs. In addition, telomere length is strongly mediated by genetic factors with an estimated heritability ranging from 44% to 80% [12], [13]. Several studies have identified a number of polymorphisms that were associated with telomere length [8], [14]–[17], but a comprehensive understanding of the genetic contribution to telomere length has still not emerged.

We recently conducted a genome-wide association study (GWAS) of lung cancer among never-smoking females in Asia and demonstrated that the rs2736100 polymorphism in the *TERT-CLPTM1L* locus on chromosome 5p15.33 was strongly and significantly associated with risk of adenocarcinoma of the lung (P trend = 10^−20^) and is replicated by other GWAS studies [18]–[20]. Further, there is additional evidence suggesting that this locus is associated with other cancers, including cancer of the bladder, prostate, uterine cervix, pancreas, breast, ovary, testes, brain, and skin [21]–[26]. These findings suggest that the *TERT-CLPTM1L* 5p15.33 region could play a role in the development of a wide spectrum of cancers with differing etiologies.

We previously reported that longer telomere length was associated with risk of lung cancer in the Alpha-Tocopherol, Beta-Carotene Cancer Prevention (ATBC) prospective cohort [4], which is comprised of smoking males in Finland. To follow up on this report in a distinctly different population and to further explore our genetic findings, we conducted a nested case-control study of lung cancer cases and controls in the prospective Shanghai Women’s Health Study cohort (SWHS).

## Materials and Methods

### Study Subjects

The detailed methods for the SWHS have been described previously [27]. Briefly, 74,942 Chinese women between the ages of 40 and 70 years and residing in seven urban communities of Shanghai were recruited into the cohort study from 1997 to 2000 with a participation rate of 92.7%. Of the study participants, 56,831 (75.8%) provided a blood sample, which was collected during enrollment into the cohort. The study was approved by the institutional review boards of all collaborating institutions. Each study subject provided a consent form at the time of enrollment and completed a standardized questionnaire including information on demographic characteristics, medical history, family history of cancer, tobacco use, residential history including use of cooking oil and fuel, and exposure to environmental tobacco smoke from their husband or from colleagues in the workplace. Follow-up for cancer incidence and mortality was conducted through home visits as well as linkage to the population-based Shanghai Cancer Registry. For women who were diagnosed with cancer, medical charts were reviewed and characteristics of the tumor were recorded. For the follow-up surveys, the follow-up rates for three different interviews were 99.8% (2000–2002), 98.7% (2002–2004), and 96.7% (2004–2007) of cohort members or their next of kin.

We used a nested case-control design for this project. The cases consist of incident lung cancer cases. Controls were selected from the SWHS study participants who were alive and free of cancer at the time of the case diagnosis, and were individually matched to cases on date of birth (±2 years) and date of blood sample collection (±3 month). In total, 215 lung cancer cases and 215 matched controls were included in the current study.

### Telomere Assay

DNA was extracted from buffy coats using the phenol-chloroform method. A monochrome multiplex quantitative PCR assay was used to determine the telomere measurements [28]. All assays were carried out at the laboratory of Dr. Richard Cawthon using the Bio-Rad CFX384 Real-Time PCR Detection System. Masked replicate samples were interspersed within and across assay batches to evaluate assay reproducibility. Cases and their matched controls were assayed consecutively within batches. The overall coefficient of variation (CV) of replicate samples was 11% and intraclass correlation coefficient (ICC) was 87%. In brief, the reagents in the 10 µL PCR were 10 mmol/L Tris-HCl (pH 8.3), 50 mmol/L KCl, 3 mmol/L MgCl2, 0.2 mmol/L each dNTP, 1 mmol/L DTT, 1 mol/L betaine, 0.75× SYBR Green I, and AmpliTaq Gold DNA polymerase, 0.625 U. The four primers were (5′ to 3′): telg (at 100 nmol/L), ACACTAAGGTTTGGGTTTGGGTTTGGGTTTGGGTTAGTGT; telc (at 900 nmol/L), TGTTAGGTATCCCTATCCCTATCCCTATCCCTATCCCTAACA; albugcr2 (at 700 nmol/L), CGGCGGCGGGCGGCGCGGGCTGGGCGGCCATGCTTTTCAGCTCTGCAAGTC; and albdgcr2 (at 500 nmol/L), GCCCGGCCCGCCGCGCCCGTCCCGCCGAGCATTAAGCTCTTTGGCAACGTAGGTTTC.

From 5 to 10 ng of human genomic DNA were added per reaction well. Three-fold serial dilutions of a reference genomic DNA sample were used to generate two standard curves for each PCR plate (five concentrations with a high of 40 ng/reaction and a low of 0.49 ng/reaction). Thermal cycling consisted of 1 cycle of 15 min at 95°C; 2 cycles of 2 s at 98°C, 30 s at 49°C; 36 cycles of 2 s at 98°C, 30 s at 59°C, 15 s at 74°C with signal acquisition, 30 s at 84°C, and 15 s at 85°C with signal acquisition. The 74°C reads provided the cycle thresholds (Cts) for telomeres; the 85°C reads provided the Cts for the single copy gene (albumin). After the run was complete, the Bio-Rad CFX Manager Software was used to determine the T (telomere) and S (single copy gene) values for each experimental sample by the Standard Curve method.

All samples were assayed in triplicate. The ratio of the telomere PCR signals to the single copy gene (in our case, β-globin) PCR signal (i.e. the T/S ratio) is proportional to the average telomere length of all the cells in a sample. T/S values are relative average telomere lengths, expressed relative to the T/S value of the reference standard DNA sample, which by definition is 1.00. The standard DNA sample used for our studies has an actual average telomere length of approximately 3300 base pairs [28]. For a given experimental sample, the T value is the number of nanograms of the reference DNA that matches the experimental sample for copy number of the telomere template, and the S value is the number of nanograms of the reference DNA that matches the experimental sample for copy number of the single copy gene template. T/S, therefore, is a relative and dimensionless value. Samples with a T/S>1.0 have an average telomere length greater than that of the standard DNA; samples with a T/S<1.0 have an average telomere length shorter than that of the standard DNA. Multiplex quantitative PCR eliminates a major source of variation present in monoplex quantitative PCR. In monoplex quantitative PCR, variation in the amount of DNA pipetted into the T and S reaction wells results in variation in T/S whereas in multiplex quantitative PCR both T and S are measured in each reaction well, so the pipetting variation between wells does not affect T/S.

### Genotyping

Genotyping was conducted using an optimized TaqMan assay (ABI, Foster City, CA), at the National Cancer Institute Cancer Genomics Research (CGR) as previously described [19].

### Statistical Analysis

Because the T/S ratios derived from the telomere length data were not normally distributed, the data were log transformed. The Wilcoxon signed-rank test was used to test the difference of telomere length among cases versus controls. The cut points for the tertiles of telomere length were derived from the distribution in the controls. Odds ratios (ORs) and 95% confidence intervals (95% CIs) were estimated using conditional logistic regression models. Telomere length was modeled as both a continuous and categorical variable. Tests for trends were calculated using log transformed telomere length as a continuous variable. Age was used in all conditional models and account for potential residual confounding, and smoking status was included in all models that included ever-smokers. Variables that resulted in a 10% or greater change in the β-coefficient of the telomere length variable in the base model were considered confounders and included in the final, multivariable models. All P values are 2-sided. We also conducted a secondary analysis using fractional polynomials to investigate a possible non-linear association between telomere length and risk of lung cancer. The optimal degree of smoothing was chosen using a model selection procedure proposed by Royston and Sauerbrei [29]. To determine if the association might be driven in part by elevated telomere length among cases with undiagnosed lung cancer at the time of blood sample collection, and to determine if the association persisted for more than 5 years, we stratified the analyses by time from blood collection to case diagnosis (0−< = 2, >2–5, and >5 years) or reference date that the control was selected. We analyzed the influence of the rs2736100 genotype on log-transformed telomere length by linear regression, assigning the ordinal values 1, 2, and 3 for the TT, GT, and GG genotypes, respectively, and adjusting for age.

## Results

The baseline characteristics of the study subjects are shown in Table 1. The age of cases and controls was comparable and 93% of cases and 95% of controls were never-smokers. Telomere length was inversely correlated with age in both cases (Spearman correlation r = −0.39 P<0.0001) and controls (Spearman correlation r = −0.41 P<0.0001). No significant correlation was found between the mean telomere length and demographic parameters shown in Table 1. In addition, the distribution of telomere length was statistically significantly longer among cases than controls [P = 0.032].

**Table 1:** Selected characteristic of lung cancer cases and individually matched controls selected from the Shanghai Women’s Health Study (recruited between 1997–2000).

Characteristic	Controls (N = 215)		Cases (N = 215)	
	N	(%)	N	(%)
Age at enrollment, y$				
40–44	16	(7.44)	15	(6.98)
45–49	23	(10.70)	25	(11.63)
50–54	23	(10.70)	24	(11.16)
55–59	26	(12.09)	26	(12.09)
60–64	44	(20.47)	47	(21.86)
65+	83	(38.60)	78	(36.28)
Ever Smoking£				
Yes	10	(4.65)	16	(7.44)
No	205	(95.35)	199	(92.56)
Passive Smoking£				
Yes	161	(74.88)	153	(71.16)
No	33	(15.35)	36	(16.74)
NA†	21	(9.77)	26	(12.09)
Family history of lung cancer‡				
Yes	1	(0.47)	3	(1.40)
No	214	(99.53)	212	(98.60)
Year of enrollment				
1997	61	(28.37)	53	(24.65)
1998	97	(45.12)	108	(50.23)
1999	47	(21.86)	42	(19.53)
2000	10	(4.65)	12	(5.58)
Lung cancer histologic subtype				
Adenocarcinoma			93	(43.26)
Other/NOS*			122	(56.74)
rs2736100				
GG	24	(11.16)	41	(19.07)
GT	103	(47.91)	109	(50.70)
TT	70	(32.56)	43	(20.00)
NA††	18	(8.37)	22	(10.23)

$ Spearman correlation (r) with telomere length in controls is −0.41(P<0.0001).

£ P value of spearman r with telomere length in controls >0.05.

‡ Family history of lung cancer in first degree relatives.

* NOS indicates not otherwise specified.

† NA indicates not available.

†† NA indicates not available, as only never-smoking subjects were genotyped.

The risks of lung cancer by tertiles of telomere length are shown in Table 2. Subjects in the middle and highest tertiles had higher risk than did those in the lowest tertile (OR 1.4, 95% CI 0.8–2.5 for the middle tertile; 2.2, 1.2–4.0 for the highest tertile, P trend = 0.003, Table 2, adjusted for age and ever smoking). Further adjustment for passive smoking and family history of lung cancer had a negligible impact on the results (OR 1.4, 95% CI 0.8–2.4 and 2.2, 1.2–4.0 for the middle and highest tertiles, respectively, P trend = 0.004). Removal of ever-smoking cases and controls yielded similar results (OR 1.7, 95% CI 0.9–2.9 for the middle tertile; 2.5, 1.3–4.7 for the highest tertile, P trend = 0.003). Models including fractional polynomials did not fit the data better than the simpler linear model (likelihood ratio test p = 0.44). The latter was thus retained on the ground of parsimony. The observed associations were similar across different follow-up times (Table 2) (i.e., time from date of phlebotomy to date of diagnosis), and a test of heterogeneity for associations across strata of follow-up time was not significant (P = 0.15). However, due to the small number of subjects in some strata and the imprecise risk estimates, replication in studies with larger samples sizes is needed to evaluate potential heterogeneity. In addition, there was no evidence that the association between telomere length and risk of lung cancer varied by age of blood donation (test for heterogeneity: P = 0.96).

**Table 2:** Telomere length and risk of lung cancer: results for overall study and stratifying by years from enrollment to case diagnosis.

Telomere Length†	Overall		Age from enrollment to case diagnosis					
			≤2		>2–5		>5	
	N_Co_/N_Ca_ *	OR(95%CI)£	N_Co_/N_Ca_ *	OR(95%CI)£	N_Co_/N_Ca_ *	OR(95%CI)£	N_Co_/N_Ca_ *	OR(95%CI)£
<1.37	71/54	1.0	12/10	1.0	26/21	1.0	33/23	1.0
1.37–1.60	72/69	1.4(0.8–2.5)	13/8	0.6(0.1–3.0)	32/26	1.3(0.6–3.2)	27/35	2.6(1.0–6.5)
≥1.60	72/92	2.2(1.2–4.0)	12/19	4.1(0.7–25.1)	33/44	2.5(0.9–7.1)	27/29	2.3(0.9–5.8)
P trend	0.003‡		0.16‡		0.069‡		0.076‡	

† Telomere length categorized using tertiles in controls as cut-points.

* N_Co_ indicates number of controls; and N_Ca_, number of cases.

£ Odds ratios computed using conditional logistic regression adjusted for age and ever smoking.

‡ P trend calculated by using log transformed telomere length as continuous variable, adjusted for age and ever smoking.

We previously reported that rs2736100 on chromosome 5p15.33 was strongly associated with risk of adenocarcinoma of the lung among never-smokers in this cohort. We therefore studied the relationship between the genetic data and telomere length in this population. The mean telomere length in association with rs2736100 (*CLPTM1L-TERT*) is shown in Table 3. The rs2736100 G allele, which we previously demonstrated to be associated with risk of lung cancer in this cohort [19], was significantly associated with longer telomere length (P trend = 0.03) (Table 3).

**Table 3:** Mean telomere length in association with rs2736100 (*CLPTM1L-TERT*), by case-control status in the Shanghai Women’s Health Study*(All cases and controls).

			Telomere length		
Genotype	Controls(%) (%)	Cases (%)	All‡	Cases‡	Controls‡
TT	70 (36)	43 (22)	1.45(0.30)	1.50(0.34)	1.43(0.28)
GT	103 (52)	109 (56)	1.53(0.29)	1.56(0.30)	1.50(0.29)
GG	24 (12)	41 (21)	1.55(0.33)	1.57(0.37)	1.52(0.25)
P trend†			0.030	0.20	0.20
Correlation P value£			0.035	0.38	0.11

* log transformed telomere length as continuous variable was used.

‡ mean (SD).

† P for trend calculated by using linear regression and rs2736100 by assigning the ordinal values 1, 2, and 3 for TT, GT, and GG respectively, adjusted for age and ever smoking.

£ P value from spearman correlation test.

## Discussion

In this prospective cohort study of mostly never-smoking women in China, we found that longer telomere length measured in peripheral white blood cells was significantly and positively associated with increased risk of lung cancer and this association was unchanged by the length of time from blood collection to case diagnosis. Further, the rs2736100 G allele, which we previously demonstrated to be associated with risk of lung cancer in this cohort [19], was also significantly associated with longer telomere length.

Our finding that longer telomere length is associated with lung cancer risk is consistent with our previous report in the prospective ATBC Cancer Prevention cohort [4] in male smokers. Both of the two studies are prospective by design and are the only cohort studies of telomere length and lung cancer published to date, to the best of our knowledge. In contrast, some case-control studies have reported associations between shorter telomere length and lung cancer risk [30]–[32] where telomere length was measured in white blood cells collected at the time of or after diagnosis of cancer. It is interesting that a report from the same cohort found a U-shaped association between telomere length and colorectal cancer risk, suggesting that the relationship between telomere length measured in peripheral blood leukocytes and risk of various types of cancers may exhibit different types of dose-response relationships.

The inconsistency between findings in case-control and cohort studies may be due to a number of reasons. First, telomere length in peripheral white blood cells could be altered by the presence of malignant disease. Therefore, the observed association between shortened telomere length and disease in case-control studies could be a result of reverse causation bias [16], [33]. Secondly, chemotherapy or radiation therapy prior to blood collection could cause DNA damage and might affect telomere length [34], [35]. Several of the case-control studies of cancer did not report the status of the treatment of the cases. Pooley et al. compared results of telomere length from samples of both retrospective case-control studies and prospective cohort studies of breast and colon cancer patients and found that shorter telomere length was associated with increased risk in the case-control studies but not in the prospective cohort studies [16], [33]. Blood samples from subjects in our study were collected one to 10 years prior to diagnosis, and the effects we report persisted for the longest period of follow-up (i.e., more than 5 to 10 years). Third, accuracy and precision in the measurement of telomere length is critical in calculating risk of cancer [36] and variation in these may contribute to inconsistent findings. For example, plate and location of sample on a plate had a significant influence on telomere length measurements even though they are highly correlated between plates within a given individual [36]. The monochrome multiplex quantitative PCR method used in this report, which was developed by Dr. Richard Cawthon [28], provides improved consistency compared to other methods [8]. In our study, Dr. Cawthon’s laboratory conducted the analyses with a coefficient of variation (CV) of 11%. Also, we enhanced precision in the measurement of telomere length by putting DNA from cases and their matched controls next to each other on a given plate.

In principle, either long or short telomeres may raise cancer risks, depending on the cell’s history of somatic mutations and the cell’s microenvironment. When the cell cycle checkpoint, cellular senescence, and apoptosis gene networks are intact, short telomeres in dividing cells are expected to be protective against cancer, since further telomere shortening caused by cell division soon triggers either cellular senescence or apoptosis, preventing the cell from developing into a cancer cell. Conversely, long telomeres may raise cancer risk, because the additional rounds of cell division afforded by the long telomeres provide the cell (and its daughter cells) the opportunity to accumulate genetic hits (somatic mutations) that block the pathways to senescence and apoptosis and promote tumorigenesis. Alternatively, among cells in which the pathways to cellular senescence and apoptosis are already blocked, those with shorter telomeres are prone to end-to-end chromosome fusions and consequent chromosomal instability, a contributor to carcinogenesis [37], [38]. A previous study suggested that a balance between elongation by telomerase and telomere shortening is important to produce a stabilized ‘optimal’ length that is critical for cell proliferation, senescence, and control [39].

The finding of an association between the *TERT* SNP rs2736100 and telomere length is intriguing. Rafnar et al. found that two SNPs on the *TERT* gene, rs401681C and rs2736098A alleles, were both significantly associated with shorter telomere length in never-smoking women between 85 and 95 years old in Caucasians. However, these associations went in the opposite direction when limited to younger never-smoking women although the associations were not significant [23]. Mirabello et al. found no association between rs2736100 and telomere length in Caucasians [40]. We note that the rs2736100 and lung cancer association was stronger among never-smoking women in East Asians than in Caucasians [19], [41]. Further, the risk was also higher compared to a GWAS of lung cancer in Japanese and Koreans, who were mostly smokers and male [42]. As such, it is possible that the relationship between rs2736100 and telomere length itself may vary by ethnicity, gender, and/or smoking behavior. Interestingly, in a preliminary analysis that was limited by small numbers, we found some evidence that the effect of longer telomere length and greater risk of lung cancer was limited to those individuals carrying the at risk (GT/GG) genotype of rs2736100 (data not shown). At the same time, our study is relatively small and the findings need to be evaluated in additional populations, using telomere length measured by the same method used here. Further, functional work is needed to identify the variant that directly accounts for the underlying association as well as to study the mechanism by which this genetic variant mediates telomere length.

In summary, our prospective cohort study found that longer telomere length measured in peripheral white blood cell DNA was strongly associated with risk of lung cancer in Chinese women, and is the second cohort study to report an association between longer telomere length and higher risk of lung cancer. However, these findings are based on relatively modest sized samples and require replication in additional cohorts and populations.
